# Draft Genome Sequence of a Psychrophilic Bacterium, *Sphingomonas antarcticum*, Isolated from the Soils of Schirmacher Oasis, Antarctica

**DOI:** 10.1128/genomeA.00696-14

**Published:** 2014-10-02

**Authors:** Ara Sreenivas, Gundlapally Sathyanarayana Reddy, Sisinthy Shivaji

**Affiliations:** CSIR-Centre for Cellular and Molecular Biology, Habsiguda, Hyderabad, Telangana, India

## Abstract

We report the 4.5-Mbp genome sequences of *Sphingobacterium antarcticum* 4BY, a psychrophilic bacterium isolated from the soils of Schirmacher Oasis, Antarctica. The draft genome of *S. antarcticum* strain 4BY consists of 4,566,318 bp with 40.4% G+C content, 4,234 protein coding genes, and 52 RNAs.

## GENOME ANNOUNCEMENT

*Sphingobacterium antarcticum* 4BY is a Gram-negative, non-spore-forming, straight-rod, nonmotile bacterium isolated from the soils of Schirmacher Oasis, Antarctica (1). Strain *S. antarcticum* 4BY is phylogenetically, based on the 16S rRNA gene sequence, closely related to the species *Pedobacter piscium* (DSM11725) with a sequence similarity of 99.9%. The genome of *S. antarcitum* strain 4BY was sequenced using Roche 454 (FLX Titanium) pyrosequencing platform. The sequencing yielded 58,988,076 bases in 182,303 reads, which is 14× coverage of the genome. The assembly of the raw sequencing data was performed using GS De Novo Assembler (version 2.8; F. Hoffmann-La Roche Ltd., Switzerland). Assembly yielded 122 contigs, all of which were ≥552 bp, with the largest contig being 323,927 bp in length; 4,566,318 bp (99.54%) were above the quality score of 40, with an average quality score of 63.69. The G+C content of *S. antarcticum* strain 4BY was calculated to be 40.4%. The genome annotation was performed using RAST (Rapid Annotation using Subsystem Technology) (2), tRNA was predicted by tRNAscan-SE (v1.23) (3), and rRNA genes were predicted by RNAmmer (v1.2) (4).

The annotation has 4,234 predicted genes, including one 5S rRNA, one 23S rRNA, one 16S rRNA, and 49 tRNA genes. In the annotation there are 105 genes encoding for resistance against antibiotics and toxic compounds including 6 genes for arsenic resistance, 2 genes for zinc resistance, 12 genes for β-lactamase resistance, 4 genes for fluoroquinolones resistance, 52 genes for cobalt-zinc-cadmium resistance, and 17 genes for copper homeostasis. There are 121 genes for membrane transport including 92 Ton and Tol transport systems. Several stress response genes (79 genes) were also predicted in the annotation.

The functional comparison of the genome sequence using the SEED framework for comparative genomics (http://www.theseed.org) revealed the closest neighbors of *S. antarcticum* strain 4BY to be *Pedobacter* sp. BAL39, *Pedobacter heparinus* DSM 2366, *S. spiritivorum* ATCC 33861, and *Algoriphagus* sp. PR1, with similarity score values of 541, 534, 366, and 326, respectively.

The whole-genome sequencing of *S. antarcticum* strain 4BY, isolated from the soils of Schirmacher Oasis, Antarctica, will help our understanding of the adaptation of psychrophiles to survive and proliferate at extremely low temperatures.

### Nucleotide sequence accession numbers.

The draft genome sequence of *S. antarcticum* strain 4BY has been deposited in DDBJ/EMBL/GenBank under the accession no. JNFF00000000. The version described in this paper is the first version.

## References

[1] Shivaji S Ray MK Shyamala Rao N Saisree L Jagannadham MV Seshu K Reddy GS Bhargava PM 1992 Sphingobacterium *antarcticus* sp. nov., a psychrotrophic bacterium from the soils of novel bacterium of the family *Sphingobacteriaceae* phylum *Bacteroidetes* isolated from Arctic soil. Int. J. Syst. Evol Microbiol. 42:102–106. 10.1099/00207713-42-1-102

[2] Aziz RK Bartels D Best AA DeJongh M Disz T Edwards RA Formsma K Gerdes S Glass EM Kubal M Meyer F Olsen GJ Olson R Osterman AL Overbeek RA McNeil LK Paarmann D Paczian T Parrello B Pusch GD Reich C Stevens R Vassieva O Vonstein V Wilke A Zagnitko O 2008 The RAST server: Rapid Annotations using Subsystems Technology. BMC Genomics 9:75. 10.1186/1471-2164-9-75 18261238 PMC2265698

[3] Lowe TM Eddy SR 1997 tRNAscan-SE: a program for improved detection of transfer RNA genes in genomic sequence. Nucleic Acids Res. 25:955–964. 10.1093/nar/25.5.0955 9023104 PMC146525

[4] Lagesen K Hallin P Rødland EA Staerfeldt HH Rognes T Ussery DW 2007 RNAmmer: consistent and rapid annotation of ribosomal RNA genes. Nucleic Acids Res. 35:3100–3108. 10.1093/nar/gkm160 17452365 PMC1888812

